# Exploring the Histone Acetylation Cycle in the Protozoan Model *Tetrahymena thermophila*

**DOI:** 10.3389/fcell.2020.00509

**Published:** 2020-06-30

**Authors:** Suzanne Wahab, Alejandro Saettone, Syed Nabeel-Shah, Nora Dannah, Jeffrey Fillingham

**Affiliations:** Department of Chemistry and Biology, Ryerson University, Toronto, ON, Canada

**Keywords:** histone acetyltransferase, bromodomain, chromatin remodeling, histone deacetylase *Tetrahymena thermophila*, *Tetrahymena*

## Abstract

The eukaryotic histone acetylation cycle is composed of three classes of proteins, histone acetyltransferases (HATs) that add acetyl groups to lysine amino acids, bromodomain (BRD) containing proteins that are one of the most characterized of several protein domains that recognize acetyl-lysine (Kac) and effect downstream function, and histone deacetylases (HDACs) that catalyze the reverse reaction. Dysfunction of selected proteins of these three classes is associated with human disease such as cancer. Additionally, the HATs, BRDs, and HDACs of fungi and parasitic protozoa present potential drug targets. Despite their importance, the function and mechanisms of HATs, BRDs, and HDACs and how they relate to chromatin remodeling (CR) remain incompletely understood. *Tetrahymena thermophila* (Tt) provides a highly tractable single-celled free-living protozoan model for studying histone acetylation, featuring a massively acetylated somatic genome, a property that was exploited in the identification of the first nuclear/type A HAT Gcn5 in the 1990s. Since then, *Tetrahymena* remains an under-explored model for the molecular analysis of HATs, BRDs, and HDACs. Studies of HATs, BRDs, and HDACs in *Tetrahymena* have the potential to reveal the function of HATs and BRDs relevant to both fundamental eukaryotic biology and to the study of disease mechanisms in parasitic protozoa.

## Introduction/Background

### Chromatin Remodeling

Eukaryotic cells package their genomic DNA into chromatin. The basic unit of chromatin, the nucleosome, consists of a histone octamer of four core histones: H2A, H2B, H3, and H4 (Luger et al., 1997). Histone variants are able to substitute for the core canonical histones within the nucleosomes and often confer specific structural and functional features (Henikoff and Smith, 2015). Additional factors, such as linker histones, further organize nucleosomes into higher-order chromatin structures (Fyodorov et al., 2018). Chromatin ultimately needs to be remodeled for DNA transactions such as transcription to occur (Clapier and Cairns, 2009). Mechanisms of chromatin remodeling (CR) involve ATP-dependent histone sliding [e.g., SWI/SNF (Johnson et al., 2005), INO80 (Poli et al., 2017), and ISWI (Yan et al., 2016)] and the selective insertion/removal of histone variants [SWR (Morrison and Shen, 2009) and INO80 (Brahma et al., 2017)], as well as the post-translational modification (Gardner et al., 2011) (PTM) of specific amino acids within histones including lysine acetylation and methylation. Histone PTMs can lead to downstream events via recruitment of proteins with specific PTM-interacting domains including the bromodomain (BRD) that interacts with acetylated lysines (Filippakopoulos et al., 2012).

### The Histone Acetylation Cycle and Its Relevance to Human Disease

The histone acetylation cycle begins with the selective addition of an acetyl group to a specific lysine residue in histones, a process known as histone acetylation, and is catalyzed, or “written,” by histone acetyltransferases [HATs (Berndsen and Denu, 2008)]. HATs can be guided to their specific histone targets by physically interacting with proteins containing histone-interacting domains (Lalonde et al., 2014). Bromodomain proteins are able to interact, or “read” acetyl-lysine (Kac), and the cycle is complete when the acetyl is removed, or “erased,” by histone deacetylases (HDACs) (Kuo and Allis, 1998). Histone acetylation occurs either at the nucleosomal level by type A HATs [SAGA (Baker and Grant, 2007) and NuA4 (Doyon and Cote, 2004) complexes] or on histones prior to their deposition into chromatin by type B HATs [Hat1 (Parthun, 2007), Rtt109 (Fillingham et al., 2008)]. Although the focus of this review is histone acetylation, it is important to note that proteomic studies have identified hundreds to thousands of acetylated proteins in a variety of model systems from parasitic protozoa to mammalian cells (Zhao et al., 2010; Jeffers and Sullivan, 2012; Miao et al., 2013; Li et al., 2019). To reflect this, HATs and HDACs that acetylate/deacetylate the group of lysine residues on non-histone substrates are also be referred to as lysine acetyltransferases/deacetylases (KATs/KDACs) (Allis et al., 2007).

The proteins of the histone acetylation cycle have clinical significance in cancer (Somech et al., 2004; Jain and Barton, 2017; Richters and Koehler, 2017) and have attracted interest as potential druggable targets. For example, translocation of BRD-containing BRD4 to NUTM1 in human cells generates an oncoprotein that drives a rare and aggressive form of squamous cell carcinoma, NUT midline carcinoma (NMC) (French et al., 2004). Multiple small molecules have been developed that disrupt BRD-Kac interactions (Cochran et al., 2019) and are subject of investigation for their efficacy in treating cancers such as NMC. HATs, BRDs, and HDACs are often required for the viability and pathogenesis of parasitic protozoa (Jeffers et al., 2017) which are among the Neglected Tropical Diseases (NTD) common in regions of Africa, Asia, and the Americas (World Health Organization, 2010). Anti-protozoa drugs exist but their efficacy is being compromised by rising resistance (de Koning, 2017). The availability of small molecule inhibitors to proteins of the histone acetylation cycle has driven interest in their use to treat these diseases. The Alveolate lineage of eukaryotes includes the parasitic apicomplexa, with *Plasmodium* and *Toxoplasma* species. Gcn5 was shown to be essential for *Toxoplasma* DNA replication, prompting a search for drugs that target this HAT (Vanagas et al., 2012; Jeffers et al., 2016). In *Plasmodium*, PfBDP1 containing a C-terminal BRD and an N-terminal ankyrin repeat is required for penetration of red blood cells (Josling et al., 2015), potentially providing a drug target. Plasmodium HDACs are also being investigated as possible drug targets (Andrews et al., 2012).

### *Tetrahymena thermophila* as a Model for the Study of the Histone Acetylation Cycle

**FIGURE 1:**
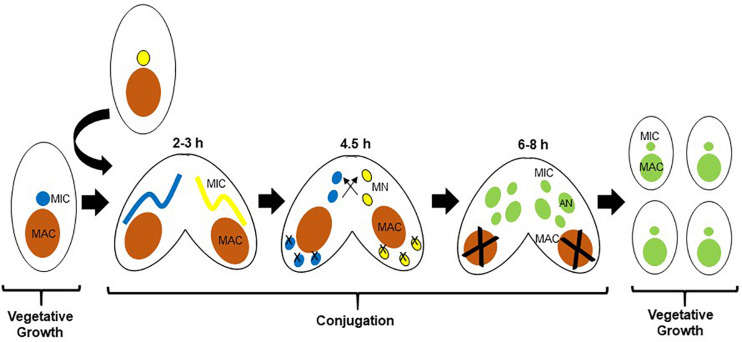
Life cycle of *Tetrahymena thermophila* (Tt). Parental macronuclei are shown in orange. After pairing of two cells of different mating types, the respective MICs (shown in blue and yellow) undergo meiosis. In each cell, one of the four meiotic products is selected as the nucleus that will be inherited, and the other three degenerate. The selected meiotic product in each cell undergoes mitosis to produce two identical pro-nuclei, one of which is transferred to the mating partner where they fuse to form a zygotic nucleus that further divides mitotically twice to generate four nuclei (shown now in green), two of which will develop as new MACs, and two as MICs. As the new MACs are developing, the parental MAC condenses and degenerates. Time periods shown correspond to hours post mixing of the two cells.

The ciliate *Tetrahymena thermophila* (Tt), a free-living genetically tractable Alveolate, has been a beneficial model for early studies of the fundamental biology of histone acetylation due to its unique nuclear biology (Grunstein and Allis, 2018). Tt is suitable to study apicomplexan biology as well as that of other protozoan parasites due to their evolutionary relationship. Tt is also a proven model for discovery-based chromatin biology, based in part on the biology of the ciliates, which segregate germline-specific silent, and somatic transcriptionally active chromatin into two distinct nuclei (Orias et al., 2011). The micronucleus (MIC) is diploid, divides by mitosis, and is not transcribed during growth. The MIC undergoes meiosis during conjugation, the sexual phase of the life cycle, and is analogous to a germline nucleus. The macronucleus (MAC) is highly polyploid with ∼45 copies of each MAC chromosome, divides amitotically without functional centromeres, is transcriptionally active, and is not inherited sexually; analogous to a somatic nucleus. During amitosis, multiple copies of each macronuclear chromosome are randomly partitioned between the two daughter cells. As a result, alleles segregate randomly, and therefore vegetative progeny of a cell initially heterozygous after conjugation become homozygous for one of the alleles after a number of cell divisions in a process called phenotypic assortment (Orias and Flacks, 1975). The two nuclei are related to each other through the sexual life cycle, conjugation (Martindale et al., 1982), the milestones of which are shown in Figure 1. MAC development includes a variety of programmed DNA rearrangements that includes chromosome fragmentation, and programmed deletion of DNA sequence called internal eliminated sequences (IESs) which is epigenetically regulated in a process that is initiated by genome-wide transcription of non-coding RNAs (Chalker and Yao, 2001) (ncRNAs) from the normally silent MIC. The ncRNAs then direct RNAi-dependent assembly of distinct chromatin domains in the new MAC (Taverna et al., 2002; Liu et al., 2007), a prelude to DNA deletion (Yao et al., 2015) thought to be similar to the chromatin diminution seen in the parasitic nematode Ascaris (Wang et al., 2017).

*Tetrahymena thermophila* has features of a model genetic organism including fast growth in axenic culture, and the ability to undergo large scale and synchronous matings. Both the MAC (Eisen et al., 2006) and MIC (Hamilton et al., 2016) genomes have been sequenced and annotated (Stover et al., 2006). A well-developed functional proteomic pipeline exists for the study of epigenetic regulators in particular for **a**ffinity **p**urification coupled to **m**ass **s**pectrometry (AP-MS) to effectively solubilize native (i.e., un-crosslinked) protein complexes both bound and unbound to chromatin (Xiong et al., 2011; Garg et al., 2013, 2019; Saettone et al., 2018, 2019a; Ashraf et al., 2019; Nabeel-Shah et al., 2020). A critical feature of this approach is the expression of the epitope tagged proteins at levels closely approximating that of the endogenous protein. In *Tetrahymena* this is achieved by exact gene replacement mediated by homologous recombination, adding the epitope tag in-frame at the C-terminus of the endogenous protein, such that the epitope tagged polypeptide is expressed under its own promoter, as in budding yeast. Physical Interactome mapping experiments are performed using a minimum of two biological replicates in parallel to control experiments using untagged parental strains, facilitating the identification of interaction partners significantly over-represented in the samples, a process aided by use of algorithms such as SAINTexpress (Teo et al., 2014). Functional genomic approaches that have been developed for Tt include definition of the transcriptome through microarray (Miao et al., 2009) and RNA-Seq analysis (Xiong et al., 2011). Because Tt gene expression can be assessed in a variety of developmental stages, network analysis of transcriptome data can be used to predict functional relationships between genes (Xiong et al., 2011). In addition, genomic localization of proteins involved in the acetylation cycle can be tracked using ChIP-Seq (**ch**romatin **i**mmuno**p**recipitation followed by next generation **seq**uencing) (Saettone et al., 2018, 2019b).

## Histone Acetylation in *Tetrahymena thermophila*

**FIGURE 2:**
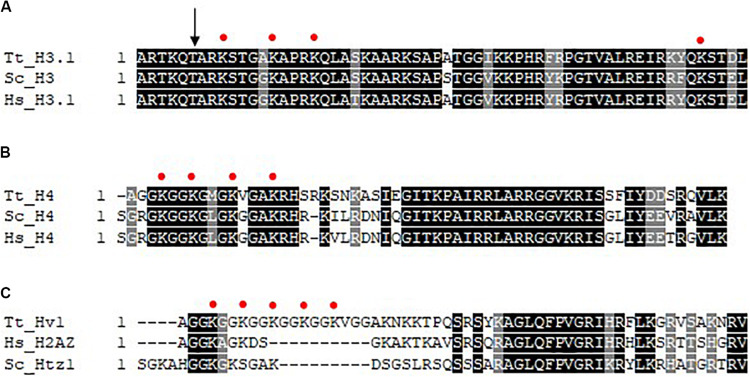
Multiple sequence alignment of histone N-termini. Clustal Omega was used to perform multiple sequence alignment (EMBL-EBI) and shaded using BoxShade (ExPASy). Absolutely conserved residues are represented by a black shade and conserved residues are represented by a gray shade. Red circles represent sites of acetylation as indicated in the text. **(A)** N-termini of histone H3. The arrow indicates site of N-terminal proteolysis. **(B)** N-termini of histone H4. **(C)** N-termini of histone H2A variant Hv1/H2AZ. Tt, *Tetrahymena thermophila*; Hs, *homo sapiens*; Sc, *Saccharomyces cerevisiae*.

In addition to their distinct morphologies and functions, differences exist in the complement of chromatin proteins as well as the degree of histone acetylation in the MAC and MIC. Although the same core histones (H2A, H2B, H3, and H4) are present in both nuclei, the MIC possesses two versions of histone H3 (Allis et al., 1979). One of which is indistinguishable from that found in the MAC (named H3S for slow), and the other is unique to MIC, and has a faster mobility in SDS-PAGE (and thus named H3F for fast) as a consequence of a regulated proteolytic event where six amino acids are removed from the N-terminus (Allis et al., 1980a; Figure 2A); the underlying enzymology of which is unknown. Nucleus-specific linker histones exist, Hho1 for the MAC and Mlh1 (Micronuclear linker histone 1) for the MIC. Mlh1 in particular is proteolyzed (Allis et al., 1984) into several smaller polypeptides (alpha, beta, gamma, and delta). Although HHO and MLH are non-essential genes, DAPI staining combined with knockout analysis showed that both function in the condensation of chromatin in their respective nuclei (Shen et al., 1995). The MIC also features the exclusive localization of CNA1 (Cervantes et al., 2006; Cui and Gorovsky, 2006), an ortholog of the centromeric-specific H3 variant CENPA, consistent with apparent lack of centromeres in the MAC. Transcription associated histone variants Hv1 (H2A.Z) and Hv2 (H3.3) are MAC-specific (Allis et al., 1980b) during growth and starvation, appearing in the MIC only during selected times in conjugation (Stargell et al., 1993; Cui et al., 2006). In addition to being widely distributed in the MAC by immunofluorescence (IF), Hv1 localizes to the nucleoli indicating that it may be involved in rDNA transcription (Allis et al., 1982). The use of specific anti-Hv1 antibodies in indirect IF indicates that Hv1 may present in the crescent MIC, a time when it is transcriptionally active (Stargell et al., 1993). Hv2 differs in 16 amino acids from the major, abundant H3 proteins and is expressed constitutively (Thatcher et al., 1994). Genetic analysis suggests that the primary importance of HHT3 in growing cells is a consequence of its constitutive expression rather than its primary sequence (Yu and Gorovsky, 1997).

Some of the earliest observations linking histone acetylation and transcription occurred using the Tt model. Pulse-labeling studies using radiolabeled acetate was used to show that histones in general are highly acetylated in the MAC but not MIC in growing cells (Vavra et al., 1982a, b), a finding that provided a key functional link of histone acetylation to gene expression. Initial studies on histone deposition-associated acetylation focused on pulse-labeling cells with radiolabeled acetate early in conjugation where MICs replicate rapidly, but are transcriptionally inactive, thus any MIC-specific histone acetylation at this time is related functionally to histone deposition and chromatin assembly. Initial studies showed that newly synthesized histone H3 and histone H4 were deposited into MIC in mono- and di-acetylated (H3) and di-acetylated (H4) forms (Allis et al., 1985). Later work identified these deposition-associated acetylation sites on K9/14 on H3 (Sobel et al., 1995; Figure 2A) and H4 K4/11 (Chicoine et al., 1986; Figure 2B, note corresponds to H4K5/12 in yeast and human cells: Tt H4 contains a single deletion at amino acid 3, so each acetylation site is numbered one less than that observed in most other H4s). Similar studies on pulse-labeled MACs isolated during growth revealed H4K7 (Figure 2B, corresponds to H4K8) and H4K4 to be the principle sites of acetylation correlated with transcription on H4, with H4K11 and H4K15 also acetylated at lower levels (Chicoine et al., 1986; Figure 2A). Transcription-associated sites of acetylation on H3 were revealed to be H3K9 and H3K14 and to a lesser extent H3K18 and H3K23 (Chicoine et al., 1986; Figure 2B). Subsequent studies found Hv1 to be acetylated at positions 4, 7, 10, 13, 16 in its N-terminus (Allis et al., 1986; Figure 2C). When indirect IF was performed with newly developed anti-tetra-acetylated H4 and anti-penta acetylated Hv1 antibodies, MAC localization was observed (Lin et al., 1989). When studying the extent of histone acetylation during conjugation, only a slight increase in acetylation was shown in the early developing new MACs compared to the developing micronuclei at the same stage. A greater amount of acetylation is detected in the advanced stage of anlagen development comparable to the parental macronuclei (Chicoine and Allis, 1986) suggesting that there is modulation of the histone acetylation cycle during nuclear development.

## Histone Acetyl-Transferases in *Tetrahymena thermophila*

Histone acetyltransferases are responsible for catalyzing the transfer of acetyl groups from acetyl-CoA onto lysine residues on the amino-termini of histones. There are five genes that encode clear orthologs of HATs [*GCN5/HAT2*, *HAT1*, and 3 MYST-family HATs (*MYST1-3*)].

### Gcn5/SAGA

The search for the gene encoding a HATs was hampered by relatively low amounts of the enzyme that made it difficult to obtain peptide sequence. For example a HAT activity was identified and characterized in yeast in the early 1980s (Travis et al., 1984), but the gene responsible was not cloned (No Author, 2018). The massive amount of acetylated chromatin in the Tt MAC was key to finding the first gene encoding a HATs. A novel SDS-PAGE acetyltransferase activity assay was used to show that the Tt MAC possess a 55 kDa protein (p55) able to incorporate [3H] acetate from [3H] acetyl-coA into a histone H3 substrate (Brownell and Allis, 1995). After partial purification using the in gel-assay to monitor the activity, Edman degradation and subsequent molecular cloning followed by comparative sequence analysis determined that p55 is orthologous to Gcn5 (Brownell et al., 1996), a transcriptional adaptor, or co-activator previously described as necessary for activity of transcriptional activators in yeast (Georgakopoulos and Thireos, 1992). This finding established the mechanistic link between chromatin structure and gene expression, and reinforced the idea of histone acetylation as a mark of transcriptionally active chromatin. Gcn5 is broadly conserved in eukaryotes present in most if not all sequenced eukaryotes including *Toxoplasma gondii* (Wang et al., 2014) and *Plasmodium falciparum* (Fan et al., 2004). In yeast and human cells, Gcn5 is found in the multi-subunit complex SAGA (Grant et al., 1997), a transcriptional co-activator complex containing ∼19 subunits (Allard et al., 1999; Daniel and Grant, 2007) that, as a type A HAT, targets the nucleosomal N-terminal tail of H3 (Grant et al., 1997, 1998, 1999). The original type A HAT, Gcn5p, may also possess type B HAT activity in *Saccharomyces cerevisiae* as it is involved in the acetylation of the NH2-terminal tail of newly synthesized histone H3 (Burgess et al., 2010).

Details on the function and mechanism of Tt Gcn5 have lagged since its initial discovery. Recombinant Tt Gcn5 acetylates free histone H3 including on H3K13 and H3K18 (Garg et al., 2013; Figure 2A). The questions as to the nature of Tt SAGA was recently addressed (Saettone et al., 2018) by AP-MS of Ibd1 (see section “Group II BRD Proteins,” below) that revealed p55/Gcn5 to be present in a complex with a clear ortholog of Ada2, as well as the BRD-containing Ibd1, and PHD-domain containing protein **A**da2-**A**ssociated **P**rotein **1** (AAP1). One function of PHD domains is to interact with methyl-lysine PTMs (Arrowsmith and Schapira, 2019). Subsequent AP-MS of Ada2 reciprocally identified Gcn5, Ibd1, Aap1 and three additional PHD domain-containing proteins (Aap2-4) that were not observed in repeated Ibd1 AP-MS. Consistent with this, a SAGA-like complex consisting of orthologs of Gcn5, Ada2, and a PHD domain protein (PHD1) in addition to a protein of unknown function, was purified from Apicomplexan *P. falciparum* by incubation of extracts with a biotinylated H3K4me3 peptide (Hoeijmakers et al., 2019). The same complex minus the protein of unknown function co-purified when extracts were incubated with an H4K5/8/12ac peptide. Additionally, AP-MS of PfGcn5 revealed an interaction with an additional PHD-domain-containing protein PHD2, purification of which enriched Gcn5 and Ada2 but not PHD1. The similarities between Gcn5 membership in multiple protein complexes in Tt and Pf suggest that multiple SAGA-like complexes exist in Alveolates that are composed of a “core” of Ada2 and Gcn5 with different epigenetic readers that in Tt and Pf are composed of PHD and BRD proteins. To demonstrate this conclusively, AP-MS of Tt Aap2-4 will be required, the prediction being that each will co-purify with Ada2 and Gcn5 but none of the other readers. If this is the case, it will be important to determine chromatin-binding specificity of the respective PHD fingers of the AAPs. BLASTP analysis indicates that AAP1 is the highest match in the Tt genome of Pf PHD1 with high homology between the two in their fourth PHD domain of PHD1 that matches consensus for H3K4me2/3-binding.

### Hat1

Hat1 was originally purified from yeast cytoplasmic extracts in a complex with Hat2 (Rbap46 in mammalian cells). Hat1 is a type B HAT, highly specific for H4K5 and H4K12 on free histones in yeast and humans (Parthun, 2007). Tt encodes a clear ortholog of *HAT1*, but to date it has not been subjected to molecular analysis. Experiments performed by Allis and colleagues showed MIC and cytoplasmic extracts of growing Tt to possess a HAT activity on Tt H4 (Richman et al., 1988) that had specificity for position 4 or positions 4 and 11 (Richman et al., 1988; Figure 2B, the sites that correspond to H4K5/12 in Tt). The same activity did not acetylate mononucleosomes, consistent with Hat1 activity in yeast and human cells (Parthun et al., 1996; Verreault et al., 1998). Based on work performed in yeast and human cells, it would make sense that this activity was performed by the Hat1 complex (Parthun et al., 1996), composed of the Hat1 HAT bound to the Hat2/RbAp46 histone chaperone (Parthun, 2013). The expression of HAT1 is essential in human cells (Nagarajan et al., 2013). Interestingly, when the cytoplasmic activity was heated to 45°C, the Hat1-like activity on H4 was retained, but now the activity also mono-acetylated H3 at an unknown lysine residue (Richman et al., 1988). It will informative to characterize the Hat1 complex in Tt and to determine whether it has activity on H3 as well as H4.

### NuA4/Esa1

NuA4/TIP60 are multi-subunit type A HAT complexes in yeast and human cells responsible for acetylation of nucleosomal histone H4 and H2A with catalytic subunit Esa1/Tip60 (Doyon and Cote, 2004; Jacquet et al., 2016). Analogous H2A/H4-specific Hat A activities are more poorly characterized outside the Opisthokonts. In Tt, a H2A/H4-specific nucleosomal HAT activity was previously partially purified from MAC DNAse-treated extracts, and labeled NuA4-like (Ohba et al., 1999). Yeast/human NuA4/TIP60 complexes and the Tt NuA4-like activity both have specificity for lysines 5, 8, 12, and 16 of H4 and lysines 5 and 9 of H2A on nucleosomes (Ohba et al., 1999), suggesting the activity could be catalyzed by an analogous Tt complex. However, the Tt NuA4-like activity co-purified on a sucrose gradient with a predicted size of ∼ 80 kD which is much smaller than that of NuA4/TIP60 complex, a 1.0–1.5 MDa multi-protein platform of at least 13–16 subunits. Comparative sequence analysis of the MAC genome suggests that there are three potential Tt genes encoding an ortholog of Esa1/Tip60, named MYST1-3, with MYST1 and MYST2 situated side by side in the MAC genome, possibly the result of a tandem duplication. Yeast also encode three MYST family HATs, each of which nucleates a distinct HAT complex with non-overlapping functions (Esa1-NuA4, Sas2 – SAS complex and Sas3 of the NuA3 complex). The parasitic protozoa *Trypanosoma brucei* (*T. brucei*) encodes three MYST family proteins named HAT1-3 that all localize to its nucleus (Kawahara et al., 2008). *P. falciparum* encodes a single gene encoding a MYST family HAT (Miao et al., 2010). Clear orthologs of genes encoding core NuA4/Tip60 proteins Eaf1 and Epl1 are not present in the Tt MAC genome or that of other Alveolates, consistent with the idea that a NuA4 complex is not well conserved outside the Opisthokonts. Identification of the Tt MYST HAT underlying the NuA4-like activity, and identification of co-purifying proteins, is likely to inform NuA4/TIP60 characterization in the parasitic protozoa.

### H3K56 Acetylation in *Tetrahymena thermophila*

H3K56ac is associated with DNA replication associated chromatin assembly, gene expression, and maintenance of genome stability in yeast and human cells (Masumoto et al., 2005; Das et al., 2009). H3K56 is conserved in Tt H3 (Figure 2A), and the MAC possesses robust levels of H3K56ac during growth (Garcia et al., 2007) and early nuclear development (Akematsu et al., 2017). Although not widely studied outside of Fungi and human cells, H3K56ac has been reported to be present in parasitic protozoa such as *P. falciparum* (Gupta et al., 2016). H3K56ac is catalyzed by Rtt109 in Fungi (Collins et al., 2007; Driscoll et al., 2007; Fillingham et al., 2008) and p300/CBP in humans (Das et al., 2009). In Fungi and human cells, histone H3/H4 chaperone ASF1 is also required to catalyze H3K56ac (Recht et al., 2006; Das et al., 2009) by the respective HAT. Tt does not encode a clear ortholog of either Rtt109 or p300/CBP but does encode a single copy of *ASF1* (Garg et al., 2013). Rtt109 has drawn recent interest as a possible drug target to combat pathogenic fungal infection due to its fungal-specific nature and importance to viability (Wurtele et al., 2010). Despite their non-homologous primary amino acid sequence, Rtt109 and p300/CBP have structural similarity of their catalytic core (Bazan, 2008). Human Gcn5 has been reported to acetylate H3K56 in human cells (Tjeertes et al., 2009), but not in yeast. Recombinant Tt p55/Gcn5 does not possess H3K56ac activity *in vitro* on core histone substrates in the presence or absence of recombinant yeast or Tt Asf1 (Garg et al., 2013) which argues against Gcn5 being the H3K56-specific HAT in Tt. It should be noted that the HAT assay was performed with chicken and not Tt histones, and that in Tt Gcn5 exists in a protein complex with an Ada2 ortholog (see section “Gcn5/SAGA”), so its behavior *in vitro* may reflect absence of key components. Functionally, the importance of the modification to growth/genome stability in Tt has yet to be determined, or even if it is linked to chromatin assembly as in yeast. Arabidopsis have also been demonstrated to encode orthologs of p300/CBP suggesting that p300/CBP was present in last common ancestor of plants and Opisthokonts, which should also include protist lineages. If H3K56ac is important to protozoan viability, the H3K56-specific HAT (particularly if novel) may have potential as a drug target for treatment of parasitic protozoa infection.

## Bromodomain Proteins in *Tetrahymena thermophila*

**FIGURE 3:**
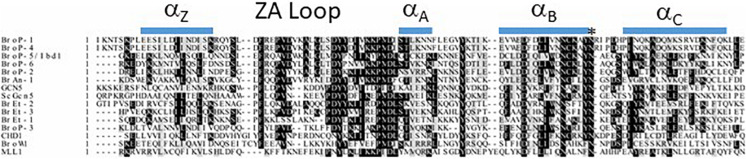
Multiple sequence alignment of 14 Tt bromodomains (BRD). BRD sequence was extracted from the respective full length sequence using SMART. Clustal Omega was used to perform multiple sequence alignment (EMBL-EBI) and shaded using BoxShade (ExPASy) with absolutely conserved residues indicated by a black shade and conserved residues by a gray shade. The asterisk represents the highly conserved Asparagine (N) that makes contact with acetyl-lysine. Predicted secondary structure (JPRED) is shown along the top of the alignment.

**FIGURE 4:**
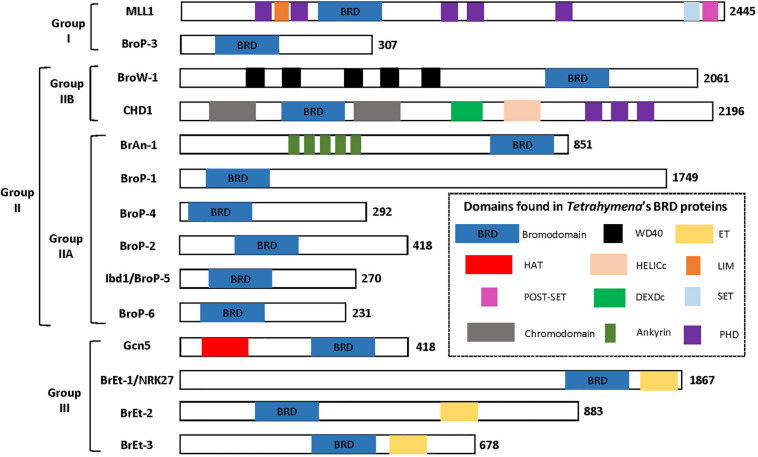
Tetrahymena’s 14 BRD proteins grouped based on phylogenetic analysis of BRD sequence. Figure adapted from Saettone et al. (2018).

There are several protein domains that selectively recognize and bind to acetylated Lysine (Kac) residues in histones (Jain and Barton, 2017) including PHD (Zeng et al., 2010), YEATS (Li et al., 2014) and the BRD that is the focus of this section. BRD-containing proteins are frequently dysregulated in cancer (Jain and Barton, 2017) and their expression has been demonstrated to be important for pathogenesis of several parasitic protozoa (Schulz et al., 2015). Importantly, BRDs can be targeted by small-molecule inhibitors leading to the idea they can be targeted to control cancer and/or infection by parasitic protozoa (Jeffers et al., 2017; Kougnassoukou Tchara et al., 2019; Hanquier et al., 2020; Nguyen et al., 2020). The polyploid MAC of Tt is massively enriched for acetylated chromatin which makes it a potentially useful system for discovering new BRD functions. A BLASTP-based survey of the Tt MAC genome revealed 14 potential BRD-containing gene products (Saettone et al., 2018). Tt BRD proteins present one BRD per protein which is different to other described eukaryotes where BRD proteins often are present in tandem. The BRD is composed of four helices with the ZA and BC loops connecting helices αZ to αA, and αB to αC. The ZA and BC loops form a hydrophobic pocket that functions in Kac binding (Dhalluin et al., 1999). The ZA and BC loops interact with residues flanking the Kac and are somewhat variable in sequence, reflecting the fact that different BRDs have distinct lysine acetylation sites in histones and non-histone proteins (Zaware and Zhou, 2019). A structure-based multiple sequence alignment of the 14 Tt BRDs (Figure 3) shows conservation of BRD secondary structure. BRD-based Kac recognition involves hydrogen bond formation with a conserved Asparagine in the BC loop (Zaware and Zhou, 2019) which is conserved in all 14 Tt BRD proteins (Figure 3). Phylogenetic analysis was performed using the 14 Tt BRD sequences (Saettone et al., 2018) permitting their delineation into three main groups which is shown along with domain analysis of the respective full length protein in Figure 4.

### Group I BRD Proteins

Group I is composed of two proteins, the first a clear ortholog of **M**ixed-**L**ineage **L**eukemia **1** (MLL1), and BroP3 (Figure 4). Mammalian MLL1 is a histone methyl-transferase (KMT2A) that positively regulates transcription by tri-methylation of H3K4 (H3K4me3) and is considered an ortholog of yeast Set1. Both mammalian MLL1 and yeast Set1 co-purify with a related protein complex, COMPASS (Shilatifard, 2012). A previous study demonstrated that hyper-acetylated MAC-specific histone H3 tends to be rich in H3K4me3 (Taverna et al., 2007). It seems possible that in Tt, highly expressed genes are rich in both modifications. Our sequence alignment indicates that the BRD of Tt MLL1 [unlike that of human (Filippakopoulos et al., 2012)] is predicted to have Kac-binding activity (Figure 3) but its relatively divergent ZA loop sequence (Figure 3) raises the possibility of a non-histone target. It is tempting to speculate that the BRD of MLL1 recruits histone methyltransferases (HMTs) to add the H3K4me3 modification to acetylated chromatin, promoting high levels of transcription. Interestingly, GroupII BRD protein Ibd1 co-purifies with another putative H3K4me3 HMT that does not itself possess a BRD (see below Section “Group II BRD Proteins”). BroP3, the other group I member, has a simple domain structure similar to Ibd1 (Section “Group II BRD Proteins”) with a single N-terminal BRD.

### Group II BRD Proteins

Group II is subdivided into two principal divisions (Figure 4). Group IIA includes chromodomain helicase protein (CHD), with BRD situated between two N-terminal chromodomains, in addition to BroW1, possessing several WD-repeats and a C-terminal BRD. The MIC copy of the gene encoding BroW1 includes an IES (Hamilton et al., 2016). IES are rarely found in MIC sequence corresponding to coding sequence in the MAC due to their imprecise mechanism of elimination.

Group IIB includes BrAn1, in addition to several proteins related by their simple domain organization, BroP1, BroP2, BroP4, BroP5, and BroP6 (Figure 4). BroP5 (also known as Ibd1 -**I**nteractive **B**R**D** protein **1**) is the best characterized BRD protein to date in Tt, initially identified as a member of the Tt SWI/SNF CR complex via AP-MS of the conserved Snf5 subunit (Saettone et al., 2018). Reciprocal AP-MS experiments revealed Ibd1 to also be a member of SWR, a SAGA-like and an HMT-containing complex (Saettone et al., 2018). Incubation of recombinant Ibd1 with a histone post-translation modification peptide array (Saettone et al., 2018) suggest that its BRD recognizes specific histone acetylation associated with the transcriptionally active MAC, H3K9/14ac, and H4K8ac (Chicoine et al., 1987; Taverna et al., 2007). Indirect IF of Ibd1 showed localization to the MAC throughout the Tt life cycle consistent with a role for Ibd1-containing CR complexes in the regulation of transcription (Saettone et al., 2018). Support for this hypothesis was provided by ChIP-Seq analysis of Ibd1 that revealed enrichment in the coding regions of highly expressed genes during growth leading to the proposal of the “pile-on” model where histone acetylation leads to Ibd1-dependent recruitment (or “piling on”) of multiple CR activities that each individually contribute to high levels of transcription. To test the model, it will be important to assess the degree to which Ibd1 recruits each of the respective CR complex to the set of Ibd1-bound highly expressed genes. Also, although the model proposes an additive effect for each CR complex, it is possible that redundancies exist among them. The CR complexes hypothesized to be recruited by the Kac-binding of Ibd1 BRD include SWR, SWI/SNF, SAGA and a putative HMT.

The putative HMT that co-purifies with Ibd1 is uncharacterized, but sequence analysis indicates that it has homology to HMTs that have specificity for H3K4 or H3K36, both modifications associated with transcription. As discussed above for MLL, the association of Ibd1 and the HMT potentially could link transcription-associated histone acetylation to H3K4me3, providing a molecular mechanism behind the observation that hyper-acetylated MAC-specific histone H3 tends to be rich in H3K4me3 (Taverna et al., 2007). The SWI/SNF complex is an ATP-dependent CR complex nucleated by SNF2/Brg1 ATPase subunit. The human Brd9 protein, similar to Ibd1 with only one BRD, was recently shown to be a member of human SWI/SNF complex (Wang et al., 2019). Unlike yeast and human cells, Tt Brg1/SNF2 does not possess a C-terminal BRD (Fillingham et al., 2006). The specificity of the Ibd1 BRD appears similar to that of the human and yeast Brg1/Snf2 subunit. The biochemical function of SWI/SNF is ATP-dependent remodeling of nucleosome structure by mobilizing nucleosomes by sliding and/or ejection of histone octamers (Saha et al., 2006). We speculate that the physical interaction of Ibd1 with SWI/SNF links histone acetylation to nucleosome sliding or ejection. Interestingly the association between Ibd1 and SWI/SNF appears much less stable during conjugation (Saettone et al., 2018) indicating that the physical interaction between the two may be subject to regulation. The function of SWR-C in yeast (SRCAP in humans) is the deposition of Htz1/H2AZ (Kobor et al., 2004; Ruhl et al., 2006). Previously the Allis lab demonstrated that Hv1, like Ibd1, localizes to the MAC of growing cells implicating Hv1 in transcription (Wenkert and Allis, 1984). Tt SWR-C was defined by the intersection of common proteins in Ibd1 and Swc4 AP-MS (Saettone et al., 2018; Ashraf et al., 2019) with clear orthologs identified for Swr1, Yaf9, Rvb1, RvB2, Swc2, Swc4, and Swc5. AP-MS of Hv1 co-purified Swr1, Swc2, Arp5, and Rvb1 (Ashraf et al., 2019), consistent with Tt SWR-C function in the deposition of Hv1. In yeast, the NuA4 HAT acetylates H4 which recruits SWR via the BRD-containing Bdf1 subunit to deposit Htz1 (Durant and Pugh, 2007; Altaf et al., 2010). It is tempting to speculate that the Tt NuA4-like activity (discussed above) acetylates H4, proving a platform for Ibd1 recruitment and subsequent Hv1 deposition. Support for this model rests in the fact that Ibd1 appears to recognize H4K8ac (Saettone et al., 2018) [analogous to H4K7ac (Figure 2B)] that is characteristic of NuA4 activity (Allard et al., 1999).

Group IIB proteins also include BroP6, BroP2, and BroP4, that are similar to BroP5/Ibd1 in that they are small (∼400 aa), with a single N-terminal BRD and no other recognizable domains. BroP6 in particular is highly similar to Ibd1/BroP5 in amino acid sequence and along with Ibd1 co-purifies in Hv1 AP-MS (Ashraf et al., 2019). INO80-C is a multi-subunit ATP-dependent CR complex (Poli et al., 2017) that possesses a histone-exchange activity that swaps nucleosomal H2A.Z/H2B with H2A/H2B (Papamichos-Chronakis et al., 2011) (in essence, the opposite of SWR-C). Several potential INO80-C subunits co-purified in AP-MS of Hv1-FZZ (Ashraf et al., 2019) including seven evolutionary conserved subunits present in INO80-C in yeast and human including INO80, Arp5, Arp8, and IES6 in addition to transcription factor YY1, a FHA domain protein and a ubiquitin hydrolase all present in human but not in yeast INO80-C. INO80-C has not been reported to possess a BRD protein in yeast but in human cells can be co-purified with Brd3 (Wai et al., 2018). Although a definitive physical link between Brop6 and Tt INO80-C awaits AP-MS of BroP6, it is tempting to speculate that while Ibd1 recruits SWR-C to deposit Hv1 into chromatin via acetylation of H4, BroP6 could regulate its removal via initial recognition of a different acetyl modification. If BroP6 is in fact found to regulate Hv1 removal, it will be important to determine the basis of Hv1 recognition. One possibility is that N-terminal Hv1 acetylation itself could be recognized by a BRD, although a previous genetic study argues that the essential function of Hv1 acetylation is mediated through a charge patch, not a *trans*-acting factor (Ren and Gorovsky, 2001).

The only BRD reported to directly recognize acetyl-H2AZ is *T. brucei* BDF2 (Yang et al., 2017). The more likely possibility is that Hv1 is recognized within an Kac nucleosomal context, analogous to how human BRD2 recognizes H2AZ-containing nucleosomes containing acetyl-H4 (Draker et al., 2012). The Allis lab used an affinity purified polyclonal anti-Hv1 antibody to argue that Hv1 (unlike Ibd1) is be present in the meiotic MIC and may function in the pathway of MIC-specific genome-wide ncRNA transcription (Martindale et al., 1985; Stargell et al., 1993). Further work will be necessary to uncover the Ibd1-indepdent mechanism by which Hv1 is deposited in the meiotic MIC, but because of the apparent absence of Ibd1, it is unlikely to be related to histone acetylation.

The last Group IIB protein BrAn1 has a domain architecture consisting of a C-terminal BRD in combination with several N-terminal ankyrin repeats, resembling that of PfBDP1 in *P. falciparum*, the knockdown of which reduced ability to penetrate red blood cells with concomitant deregulation of invasion-associated genes (Josling et al., 2015). Interactome analysis of PfBDP1 indicates that it forms a core complex with an additional BRD protein, PfBDP2 (Hoeijmakers et al., 2019) that appears to form a variety of sub-complexes with additional proteins including a PHD-domain containing protein (Hoeijmakers et al., 2019) and a DNA-binding transcription factor AP2-I (Santos et al., 2017). The domain architecture of PfBDP1, and Tt BrAn1 is also conserved in the *T. gondii* ortholog (TGME49_263580) and appears to be unique to select protist lineages including Tt and apicomplexans (Jeffers et al., 2017). Functional characterization of Tt BrAn1 should contribute to the understanding of the apicomplexan version.

### Group III BRD Proteins

Group III (Figure 4) includes p55/Gcn5 and three proteins with a single BRD in combination with an Extra-Terminal (ET) Domain. Tt p55/Gcn5 possesses a C-terminal BRD, as does Gcn5 in other organisms. A two-step model was proposed by Taverna and colleagues for yeast Gcn5 which first acetylates H3K14 to provide a platform for binding by its BRD which stimulates its HAT activity on H3K18 (Cieniewicz et al., 2014). By analogy, the role of Tt Gcn5 BRD could be similar, stimulating Gcn5 activity after initial recruitment. In this case, the role of the Ibd1 BRD within SAGA would be to recruit SAGA to a region of chromatin perhaps acetylated by the NuA4 activity described above where it would acetylate H3, stimulating transcription. Bret-1 Bret-2 and Bret-3 are predicted to have an ET domain. They are similar in domain structure to the BET sub-family of human BRD proteins (BRD2, BRD3, BRD4, and the testis-specific BRDT) that harbor at their amino-termini two BRD followed by an ET domain that mediates protein-protein interactions. BET protein are intense subjects of research in human cells where they are implicated in cancer and are targets for molecules such as JQ1. Differently to human (and yeast) BET that possess two BRDs, Tt BETs only possess one BRD.

## Histone Deacetylation in *Tetrahymena thermophila*

Histone deacetylases remove acetyl groups from lysine residues. Inhibitors that target HDACs have been used to target human diseases such as cancer (Jain and Zain, 2011; Lee et al., 2015). HDAC inhibitors are also being investigated in the treatment of parasitic diseases (Vanagas et al., 2012; Carrillo et al., 2015; Chua et al., 2017). Wiley and colleagues used a bioinformatic query of the Tt MAC genome (Smith et al., 2008) to predict the existence of 18 HDACs that are named THDs (***T****etrahymena* **H**istone **D**eacetylase) and classified according to their similarity to yeast HDACs Rpd3 (class I, 3 members including THD1), Hda1 (class II, 2 members including THD2), and Sir2 (class III, 11 members) with an additional 2 classified as HDAC-like, one of which (Thd5) is predicted to be an ortholog of HDAC11, the smallest HDAC and it is the sole member of HDAC IV family (Gregoretti et al., 2004) implicated in mitosis and meiosis (Sui et al., 2020). Detailed molecular analysis has been performed on Tt HDACs, THD1 (Wiley et al., 2000, 2005; Parker et al., 2007), and THD2 (Smith et al., 2008).

### The Class I HDAC THD1

Class I THD1 was shown to be recruited to developing new macronuclei (Wiley et al., 2000) and to be important for the integrity of macronuclear chromatin in logarithmically dividing cells (Wiley et al., 2005). Cells knocked down for *THD1* contain higher amounts of MAC DNA, large extrusion bodies, and enlarged nucleoli (Wiley et al., 2005). It was further shown (Parker et al., 2007) that MAC chromatin in *THD1* knockdowns failed to condense during starvation, which was correlated with aberrant hyper-phosphorylation of histone H1 and the overexpression of CDC2, encoding the major histone H1 kinase. Class I HDACs such as Rpd3 are conserved among eukaryotes and are frequently found in corepressor complexes, where they mediate repression by a variety of transcription factors. In humans, the SIN3/RPD3 complex that also contains RbAp46/48 in addition to several other proteins, is targeted to specific genes through protein-protein interactions between SIN3 and either DNA-binding repressors or corepressors (Lewis et al., 2004; Keogh et al., 2005). The metazoan DREAM complex is responsible for the transcriptional regulation of cell cycle-related genes (Sadasivam and DeCaprio, 2013). Recent findings hint at the existence of a DREAM complex in Tt (Zhang et al., 2018). In human cells a Sin3B/HDAC complex robustly interacts with the DREAM complex in a cell-cycle-dependent manner (Bainor et al., 2018). It remains to be determined whether THD1 exerts its phenotype though the Tt DREAM complex.

### The Class II HDAC THD2

Based on work in the 1980s, it was known that although transcription-related acetylation was never observed in the MIC, deposition-related patterns could be observed in the presence of HDAC inhibitors such as sodium butyrate (Allis et al., 1985) indicating that H3 and H4 assembled into MIC chromatin were subject to deposition-related acetylation but that was quickly removed post-assembly. GFP-tagging was used to demonstrate that the class II HDAC named Thd2 (Tt histone deacetylase 2) localized specifically to the MIC. A complete deletion of THD2 showed ectopic H3 and H4 acetylation in the MIC indicating that WT Thd2 function is to remove deposition related acetylation (Smith et al., 2008). Interestingly, the THD2 KO also displayed a defect in MIC morphology as well as the regulated proteolytic processing of its histone H3, specifically deficient in producing the fast form of H3 that in the MIC is phosphorylated on Ser10 (Allis and Gorovsky, 1981; Allis and Wiggins, 1984), a mitotic PTM necessary for chromosome condensation and segregation (Wei et al., 1998, 1999) suggesting that Thd2 functions upstream of the proteolytic cleavage and subsequent phosphorylation of Ser10 on histone H3. It will be interesting to determine if AP-MS of Thd2 can help identify the elusive protease responsible for this enigmatic process.

### Class III HDAC

Class III histone deacetylases, known as sirtuins, couple the deacetylation of lysine with the hydrolysis of NAD+ by transferring the acetyl group to the ADP-ribose moiety to form O-acetyl-ADP-ribose, releasing free nicotinamide (Dang, 2014). Nicotinamide can thus be used as an inhibitor of sirtuin class HDACs and was used to demonstrate a possible role for the sirtuins in meiotic prophase as well as the degradation of the parental MAC during conjugation (Slade et al., 2011).

More could be learned through the molecular analysis of Tt HDACs. The use of Trichostatin A, a selective inhibitor of class I and II HDACs, resulted in defects in the progression through meiosis and also affected the deletion of IESs (Duharcourt and Yao, 2002). The identity of relevant HDAC(s) that function in these processes remains unknown.

## Conclusion and Perspectives

*Tetrahymena thermophila* offers a powerful model system with well-developed functional genomics with which to explore and understand the components of the histone acetylation cycle. The unique nuclear biology of Tt has been extremely useful in the past in the development of the histone acetylation field. Despite its efficacy the model has been underexplored. A complete understanding of the function and mechanism of the Tt histone acetylation cycle, in particular the role of histone acetylation in the regulation of H2AZ dynamics, should yield fundamental knowledge on the mechanism of transcription. In addition, the position of Tt on the evolutionary tree will permit insight into Alveolate-specific biology such as the composition of NuA4 and the identity of the protist H3K56-specific HAT.

## Author Contributions

SW wrote the manuscript, edited and prepared Figure 3. AS edited the manuscript and prepared Figures 1, 4, SN-S and ND edited the manuscript. JF conceived, wrote, prepared Figure 2, and edited the manuscript. All authors read and approved the final manuscript.

## Conflict of Interest

The authors declare that the research was conducted in the absence of any commercial or financial relationships that could be construed as a potential conflict of interest.

## References

[B1] Akematsu T. Fukuda Y. Garg J. Fillingham J. S. Pearlman R. E. Loidl J. (2017). Post-meiotic DNA double-strand breaks occur in *Tetrahymena*, and require Topoisomerase II and Spo11. *eLife* 6:e26176. 10.7554/eLife.26176 PMC5482572 28621664

[B2] Allard S. Utley R. T. Savard J. Clarke A. Grant P. Brandl C. J. (1999). NuA4, an essential transcription adaptor/histone H4 acetyltransferase complex containing Esa1p and the ATM-related cofactor Tra1p. *EMBO J.* 18 5108–5119. 10.1093/emboj/18.18.5108 10487762 PMC1171581

[B3] Allis C. D. Allen R. L. Wiggins J. C. Chicoine L. G. Richman R. (1984). Proteolytic processing of h1-like histones in chromatin: a physiologically and developmentally regulated event in *Tetrahymena micronuclei*. *J. Cell Biol.* 99 1669–1677. 10.1083/jcb.99.5.1669 6208202 PMC2113348

[B4] Allis C. D. Berger S. L. Cote J. Dent S. Jenuwien T. Kouzarides T. (2007). New nomenclature for chromatin-modifying enzymes. *Cell* 131 633–636. 18022353 10.1016/j.cell.2007.10.039

[B5] Allis C. D. Bowen J. K. Abraham G. N. Glover C. V. Gorovsky M. A. (1980a). Proteolytic processing of histone H3 in chromatin: a physiologically regulated event in *Tetrahymena micronuclei*. *Cell* 20 55–64. 10.1016/0092-8674(80)90234-2 6993010

[B6] Allis C. D. Chicoine L. G. Richman R. Schulman I. G. (1985). Deposition-related histone acetylation in micronuclei of conjugating *Tetrahymena*. *Proc. Natl. Acad. Sci. U.S.A.* 82 8048–8052. 10.1073/pnas.82.23.8048 3865215 PMC391439

[B7] Allis C. D. Glover C. V. Bowen J. K. Gorovsky M. A. (1980b). Histone variants specific to the transcriptionally active, amitotically dividing macronucleus of the unicellular eucaryote, *Tetrahymena thermophila*. *Cell* 20 609–617. 10.1016/0092-8674(80)90307-4 7418000

[B8] Allis C. D. Glover C. V. Gorovsky M. A. (1979). Micronuclei of *Tetrahymena* contain two types of histone H3. *Proc. Natl. Acad. Sci. U.S.A.* 76 4857–4861. 10.1073/pnas.76.10.4857 291904 PMC413036

[B9] Allis C. D. Gorovsky M. A. (1981). Histone phosphorylation in macro- and micronuclei of *Tetrahymena thermophila*. *Biochemistry* 20 3828–3833. 10.1021/bi00516a025 7272279

[B10] Allis C. D. Richman R. Gorovsky M. A. Ziegler Y. S. Touchstone B. Bradley W. A. (1986). hv1 is an evolutionarily conserved H2A variant that is preferentially associated with active genes. *J. Biol. Chem.* 261 1941–1948. 3944120

[B11] Allis C. D. Wiggins J. C. (1984). Proteolytic processing of micronuclear H3 and histone phosphorylation during conjugation in *Tetrahymena thermophila*. *Exp. Cell Res.* 153 287–298. 10.1016/0014-4827(84)90601-3 6734746

[B12] Allis C. D. Ziegler Y. S. Gorovsky M. A. Olmsted J. B. (1982). A conserved histone variant enriched in nucleoli of mammalian cells. *Cell* 31 131–136. 10.1016/0092-8674(82)90412-3 6760982

[B13] Altaf M. Auger A. Monnet-Saksouk J. Brodeur J. Piquet S. Cramet M. (2010). NuA4-dependent acetylation of nucleosomal histones H4 and H2A directly stimulates incorporation of H2A.Z by the SWR1 complex. *J. Biol. Chem.* 285 15966–15977. 10.1074/jbc.m110.117069 20332092 PMC2871465

[B14] Andrews K. T. Tran T. N. Fairlie D. P. (2012). Towards histone deacetylase inhibitors as new antimalarial drugs. *Curr. Pharm. Des.* 18 3467–3479. 22607140

[B15] Arrowsmith C. H. Schapira M. (2019). Targeting non-bromodomain chromatin readers. *Nat. Struct. Mol. Biol.* 26 863–869. 10.1038/s41594-019-0290-2 31582844

[B16] Ashraf K. Nabeel-Shah S. Garg J. Saettone A. Derynck J. Gingras A. C. (2019). Proteomic analysis of histones H2A/H2B and variant Hv1 in *Tetrahymena thermophila* reveals an ancient network of chaperones. *Mol. Biol. Evol.* 36 1037–1055. 10.1093/molbev/msz039 30796450 PMC6502085

[B17] Bainor A. J. Saini S. Calderon A. Casado-Polanco R. Giner-Ramirez B. Moncada C. (2018). The HDAC-associated Sin3B protein represses DREAM complex targets and cooperates with APC/C to promote quiescence. *Cell. Rep.* 25 2797.e8–2807.e8. 30517867 10.1016/j.celrep.2018.11.024 PMC6324198

[B18] Baker S. P. Grant P. A. (2007). The SAGA continues: expanding the cellular role of a transcriptional co-activator complex. *Oncogene* 26 5329–5340. 10.1038/sj.onc.1210603 17694076 PMC2746020

[B19] Bazan J. F. (2008). An old HAT in human p300/CBP and yeast Rtt109. *Cell Cycle* 7 1884–1886. 10.4161/cc.7.12.6074 18583929

[B20] Berndsen C. E. Denu J. M. (2008). Catalysis and substrate selection by histone/protein lysine acetyltransferases. *Curr. Opin. Struct. Biol.* 18 682–689. 10.1016/j.sbi.2008.11.004 19056256 PMC2723715

[B21] Brahma S. Udugama M. I. Kim J. Hada A. Bhardwaj S. K. Hailu S. G. (2017). INO80 exchanges H2A.Z for H2A by translocating on DNA proximal to histone dimers. *Nat. Commun.* 8:15616. 10.1038/ncomms15616 PMC5472786 28604691

[B22] Brownell J. E. Allis C. D. (1995). An activity gel assay detects a single, catalytically active histone acetyltransferase subunit in *Tetrahymena macronuclei*. *Proc. Natl. Acad. Sci. U.S.A.* 92 6364–6368. 10.1073/pnas.92.14.6364 7603997 PMC41518

[B23] Brownell J. E. Zhou J. Ranalli T. Kobayashi R. Edmondson D. G. Roth S. Y. (1996). *Tetrahymena* histone acetyltransferase A: a homolog to yeast Gcn5p linking histone acetylation to gene activation. *Cell* 84 843–851. 10.1016/s0092-8674(00)81063-6 8601308

[B24] Burgess R. J. Zhou H. Han J. Zhang Z. (2010). A role for Gcn5 in replication-coupled nucleosome assembly. *Mol. Cell.* 37 469–480. 10.1016/j.molcel.2010.01.020 20188666 PMC2954627

[B25] Carrillo A. K. Guiguemde W. A. Guy R. K. (2015). Evaluation of histone deacetylase inhibitors (HDACi) as therapeutic leads for human African trypanosomiasis (HAT). *Bioorg. Med. Chem.* 23 5151–5155. 10.1016/j.bmc.2014.12.066 25637120

[B26] Cervantes M. D. Xi X. Vermaak D. Yao M. C. Malik H. S. (2006). The CNA1 histone of the ciliate *Tetrahymena thermophila* is essential for chromosome segregation in the germline micronucleus. *Mol. Biol. Cell* 17 485–497. 10.1091/mbc.e05-07-0698 16251352 PMC1345684

[B27] Chalker D. L. Yao M. C. (2001). Nongenic, bidirectional transcription precedes and may promote developmental DNA deletion in *Tetrahymena thermophila*. *Genes Dev.* 15 1287–1298. 10.1101/gad.884601 11358871 PMC313804

[B28] Chicoine L. G. Allis C. D. (1986). Regulation of histone acetylation during macronuclear differentiation in *Tetrahymena*: evidence for control at the level of acetylation and deacetylation. *Dev. Biol.* 116 477–485. 10.1016/0012-1606(86)90148-x 3732617

[B29] Chicoine L. G. Richman R. Cook R. G. Gorovsky M. A. Allis C. D. (1987). A single histone acetyltransferase from *Tetrahymena macronuclei* catalyzes deposition-related acetylation of free histones and transcription-related acetylation of nucleosomal histones. *J. Cell Biol.* 105 127–135. 10.1083/jcb.105.1.127 3611182 PMC2114890

[B30] Chicoine L. G. Schulman I. G. Richman R. Cook R. G. Allis C. D. (1986). Nonrandom utilization of acetylation sites in histones isolated from *Tetrahymena*. Evidence for functionally distinct H4 acetylation sites. *J. Biol. Chem.* 261 1071–1076. 3080415

[B31] Chua M. J. Arnold M. S. Xu W. Lancelot J. Lamotte S. Spath G. F. (2017). Effect of clinically approved HDAC inhibitors on Plasmodium, Leishmania and Schistosoma parasite growth. *Int. J. Parasitol. Drugs Drug Resist.* 7 42–50. 10.1016/j.ijpddr.2016.12.005 28107750 PMC5241585

[B32] Cieniewicz A. M. Moreland L. Ringel A. E. Mackintosh S. G. Raman A. Gilbert T. M. (2014). The bromodomain of Gcn5 regulates site specificity of lysine acetylation on histone H3. *Mol. Cell. Proteomics* 13 2896–2910. 10.1074/mcp.m114.038174 25106422 PMC4223480

[B33] Clapier C. R. Cairns B. R. (2009). The biology of chromatin remodeling complexes. *Annu. Rev. Biochem.* 78 273–304. 19355820 10.1146/annurev.biochem.77.062706.153223

[B34] Cochran A. G. Conery A. R. Sims R. J. III (2019). Bromodomains: a new target class for drug development. *Nat. Rev. Drug Discov.* 18 609–628. 10.1038/s41573-019-0030-7 31273347

[B35] Collins S. R. Miller K. M. Maas N. L. Roguev A. Fillingham J. Chu C. S. (2007). Functional dissection of protein complexes involved in yeast chromosome biology using a genetic interaction map. *Nature* 446 806–810. 10.1038/nature05649 17314980

[B36] Cui B. Gorovsky M. A. (2006). Centromeric histone H3 is essential for vegetative cell division and for DNA elimination during conjugation in *Tetrahymena thermophila*. *Mol. Cell. Biol.* 26 4499–4510. 10.1128/mcb.00079-06 16738316 PMC1489134

[B37] Cui B. Liu Y. Gorovsky M. A. (2006). Deposition and function of histone H3 variants in *Tetrahymena thermophila*. *Mol. Cell. Biol.* 26 7719–7730. 10.1128/mcb.01139-06 16908532 PMC1636873

[B38] Dang W. (2014). The controversial world of sirtuins. *Drug Discov. Today Technol.* 12 e9–e17. 10.1016/j.ddtec.2012.08.003 25027380 PMC4101544

[B39] Daniel J. A. Grant P. A. (2007). Multi-tasking on chromatin with the SAGA coactivator complexes. *Mutat. Res.* 618 135–148. 10.1016/j.mrfmmm.2006.09.008 17337012 PMC1892243

[B40] Das C. Lucia M. S. Hansen K. C. Tyler J. K. (2009). CBP/p300-mediated acetylation of histone H3 on lysine 56. *Nature* 459 113–117. 10.1038/nature07861 19270680 PMC2756583

[B41] de Koning H. (2017). Drug resistance in protozoan parasites. *Emerg. Top. Life Sci.* 1 627–632. 10.1042/etls20170113 PMC7289004 33525852

[B42] Dhalluin C. Carlson J. E. Zeng L. He C. Aggarwal A. K. Zhou M. M. (1999). Structure and ligand of a histone acetyltransferase bromodomain. *Nature* 399 491–496. 10.1038/20974 10365964

[B43] Doyon Y. Cote J. (2004). The highly conserved and multifunctional NuA4 HAT complex. *Curr. Opin. Genet. Dev.* 14 147–154. 10.1016/j.gde.2004.02.009 15196461

[B44] Draker R. Ng M. K. Sarcinella E. Ignatchenko V. Kislinger T. Cheung P. (2012). A combination of H2A.Z and H4 acetylation recruits Brd2 to chromatin during transcriptional activation. *PLoS Genet.* 8:e1003047. 10.1371/journal.pgen.1003047 23144632 PMC3493454

[B45] Driscoll R. Hudson A. Jackson S. P. (2007). Yeast Rtt109 promotes genome stability by acetylating histone H3 on lysine 56. *Science* 315 649–652. 10.1126/science.1135862 17272722 PMC3334813

[B46] Duharcourt S. Yao M. C. (2002). Role of histone deacetylation in developmentally programmed DNA rearrangements in *Tetrahymena thermophila*. *Eukaryot. Cell* 1 293–303. 10.1128/ec.1.2.293-303.2002 12455963 PMC118033

[B47] Durant M. Pugh B. F. (2007). NuA4-directed chromatin transactions throughout the Saccharomyces cerevisiae genome. *Mol. Cell. Biol.* 27 5327–5335. 10.1128/mcb.00468-07 17526728 PMC1952100

[B48] Eisen J. A. Coyne R. S. Wu M. Wu D. Thiagarajan M. Wortman J. R. (2006). Macronuclear genome sequence of the ciliate *Tetrahymena thermophila*, a model eukaryote. *PLoS Biol.* 4:e286. 10.1371/journal.pbio.0040286 16933976 PMC1557398

[B49] Fan Q. An L. Cui L. (2004). Plasmodium falciparum histone acetyltransferase, a yeast GCN5 homologue involved in chromatin remodeling. *Eukaryot. Cell* 3 264–276. 10.1128/ec.3.2.264-276.2004 15075257 PMC387650

[B50] Filippakopoulos P. Picaud S. Mangos M. Keates T. Lambert J. P. Barsyte-Lovejoy D. (2012). Histone recognition and large-scale structural analysis of the human bromodomain family. *Cell* 149 214–231. 10.1016/j.cell.2012.02.013 22464331 PMC3326523

[B51] Fillingham J. Recht J. Silva A. C. Suter B. Emili A. Stagljar I. (2008). Chaperone control of the activity and specificity of the histone H3 acetyltransferase Rtt109. *Mol. Cell. Biol.* 28 4342–4353. 10.1128/mcb.00182-08 18458063 PMC2447148

[B52] Fillingham J. S. Garg J. Tsao N. Vythilingum N. Nishikawa T. Pearlman R. E. (2006). Molecular genetic analysis of an SNF2/brahma-related gene in *Tetrahymena thermophila* suggests roles in growth and nuclear development. *Eukaryot. Cell* 5 1347–1359. 10.1128/ec.00149-06 16896218 PMC1539136

[B53] French C. A. Kutok J. L. Faquin W. C. Toretsky J. A. Antonescu C. R. Griffin C. A. (2004). Midline carcinoma of children and young adults with NUT rearrangement. *J. Clin. Oncol.* 22 4135–4139. 10.1200/jco.2004.02.107 15483023

[B54] Fyodorov D. V. Zhou B. R. Skoultchi A. I. Bai Y. (2018). Emerging roles of linker histones in regulating chromatin structure and function. *Nat. Rev. Mol. Cell Biol.* 19 192–206. 10.1038/nrm.2017.94 29018282 PMC5897046

[B55] Garcia B. A. Hake S. B. Diaz R. L. Kauer M. Morris S. A. Recht J. (2007). Organismal differences in post-translational modifications in histones H3 and H4. *J. Biol. Chem.* 282 7641–7655. 10.1074/jbc.m607900200 17194708

[B56] Gardner K. E. Allis C. D. Strahl B. D. (2011). Operating on chromatin, a colorful language where context matters. *J. Mol. Biol.* 409 36–46. 10.1016/j.jmb.2011.01.040 21272588 PMC3085666

[B57] Garg J. Lambert J. P. Karsou A. Marquez S. Nabeel-Shah S. Bertucci V. (2013). Conserved Asf1-importin beta physical interaction in growth and sexual development in the ciliate *Tetrahymena thermophila*. *J. Proteomics* 94 311–326. 10.1016/j.jprot.2013.09.018 24120531

[B58] Garg J. Saettone A. Nabeel-Shah S. Cadorin M. Ponce M. Marquez S. (2019). The Med31 conserved component of the divergent mediator complex in *Tetrahymena thermophila* participates in developmental regulation. *Curr. Biol.* 29 2371.e–2379.e. 31280994 10.1016/j.cub.2019.06.052

[B59] Georgakopoulos T. Thireos G. (1992). Two distinct yeast transcriptional activators require the function of the GCN5 protein to promote normal levels of transcription. *EMBO J.* 11 4145–4152. 10.1002/j.1460-2075.1992.tb05507.x 1396595 PMC556924

[B60] Grant P. A. Duggan L. Cote J. Roberts S. M. Brownell J. E. Candau R. (1997). Yeast Gcn5 functions in two multisubunit complexes to acetylate nucleosomal histones: characterization of an Ada complex and the SAGA (Spt/Ada) complex. *Genes Dev.* 11 1640–1650. 10.1101/gad.11.13.1640 9224714

[B61] Grant P. A. Eberharter A. John S. Cook R. G. Turner B. M. Workman J. L. (1999). Expanded lysine acetylation specificity of Gcn5 in native complexes. *J. Biol. Chem.* 274 5895–5900. 10.1074/jbc.274.9.5895 10026213

[B62] Grant P. A. Schieltz D. Pray-Grant M. G. Steger D. J. Reese J. C. Yates J. R. III (1998). A subset of TAF(II)s are integral components of the SAGA complex required for nucleosome acetylation and transcriptional stimulation. *Cell* 94 45–53. 10.1016/s0092-8674(00)81220-9 9674426

[B63] Gregoretti I. V. Lee Y. M. Goodson H. V. (2004). Molecular evolution of the histone deacetylase family: functional implications of phylogenetic analysis. *J. Mol. Biol.* 338 17–31. 10.1016/j.jmb.2004.02.006 15050820

[B64] Grunstein M. Allis C. D. (2018). Genetics, biochemistry, and “Simple” organisms converge to unlock secrets in histone biology: the 2018 albert lasker basic medical research award. *JAMA* 320 1233–1234. 30208392 10.1001/jama.2018.12437

[B65] Gupta D. K. Patra A. T. Zhu L. Gupta A. P. Bozdech Z. (2016). DNA damage regulation and its role in drug-related phenotypes in the malaria parasites. *Sci. Rep.* 6:23603. 10.1038/srep23603 PMC4817041 27033103

[B66] Hamilton E. P. Kapusta A. Huvos P. E. Bidwell S. L. Zafar N. Tang H. (2016). Structure of the germline genome of *Tetrahymena thermophila* and relationship to the massively rearranged somatic genome. *eLife* 5:e19090. 10.7554/eLife.19090 PMC5182062 27892853

[B67] Hanquier J. Gimeno T. Jeffers V. Sullivan W. J. Jr. (2020). Evaluating the GCN5b bromodomain as a novel therapeutic target against the parasite Toxoplasma gondii. *Exp. Parasitol.* 211:107868. 10.1016/j.exppara.2020.107868 32119930 PMC7483680

[B68] Henikoff S. Smith M. M. (2015). Histone variants and epigenetics. *Cold Spring Harb. Perspect. Biol.* 7:a019364. 10.1101/cshperspect.a019364 25561719 PMC4292162

[B69] Hoeijmakers W. A. M. Miao J. Schmidt S. Toenhake C. G. Shrestha S. Venhuizen J. (2019). Epigenetic reader complexes of the human malaria parasite, Plasmodium falciparum. *Nucleic Acids Res.* 47 11574–11588. 10.1093/nar/gkz1044 31728527 PMC7145593

[B70] Jacquet K. Fradet-Turcotte A. Avvakumov N. Lambert J. P. Roques C. Pandita R. K. (2016). The TIP60 complex regulates bivalent chromatin recognition by 53BP1 through Direct H4K20me binding and H2AK15 acetylation. *Mol. Cell.* 62 409–421. 10.1016/j.molcel.2016.03.031 27153538 PMC4887106

[B71] Jain A. K. Barton M. C. (2017). Bromodomain histone readers and cancer. *J. Mol. Biol.* 429 2003–2010. 10.1016/j.jmb.2016.11.020 27890782

[B72] Jain S. Zain J. (2011). Romidepsin in the treatment of cutaneous T-cell lymphoma. *J. Blood Med.* 2 37–47. 22287862 10.2147/JBM.S9649 PMC3262342

[B73] Jeffers V. Gao H. Checkley L. A. Liu Y. Ferdig M. T. Sullivan W. J. Jr. (2016). Garcinol Inhibits GCN5-mediated lysine acetyltransferase activity and prevents replication of the parasite *Toxoplasma gondii*. *Antimicrob. Agents Chemother.* 60 2164–2170. 10.1128/aac.03059-15 26810649 PMC4808158

[B74] Jeffers V. Sullivan W. J. Jr. (2012). Lysine acetylation is widespread on proteins of diverse function and localization in the protozoan parasite *Toxoplasma gondii*. *Eukaryot. Cell* 11 735–742. 10.1128/ec.00088-12 22544907 PMC3370464

[B75] Jeffers V. Yang C. Huang S. Sullivan W. J. Jr. (2017). Bromodomains in protozoan parasites: evolution, function, and opportunities for drug development. *Microbiol. Mol. Biol. Rev.* 81:e00047-16. 10.1128/MMBR.00047-16 PMC5312238 28077462

[B76] Johnson C. N. Adkins N. L. Georgel P. (2005). Chromatin remodeling complexes: ATP-dependent machines in action. *Biochem. Cell Biol.* 83 405–417. 10.1139/o05-115 16094444

[B77] Josling G. A. Petter M. Oehring S. C. Gupta A. P. Dietz O. Wilson D. W. (2015). A plasmodium falciparum bromodomain protein regulates invasion gene expression. *Cell Host Microbe* 17 741–751. 10.1016/j.chom.2015.05.009 26067602

[B78] Kawahara T. Siegel T. N. Ingram A. K. Alsford S. Cross G. A. Horn D. (2008). Two essential MYST-family proteins display distinct roles in histone H4K10 acetylation and telomeric silencing in trypanosomes. *Mol. Microbiol.* 69 1054–1068. 10.1111/j.1365-2958.2008.06346.x 18631159 PMC2556858

[B79] Keogh M. C. Kurdistani S. K. Morris S. A. Ahn S. H. Podolny V. Collins S. R. (2005). Cotranscriptional set2 methylation of histone H3 lysine 36 recruits a repressive Rpd3 complex. *Cell* 123 593–605. 10.1016/j.cell.2005.10.025 16286008

[B80] Kobor M. S. Venkatasubrahmanyam S. Meneghini M. D. Gin J. W. Jennings J. L. Link A. J. (2004). A protein complex containing the conserved Swi2/Snf2-related ATPase Swr1p deposits histone variant H2A.Z into euchromatin. *PLoS Biol.* 2:E131. 10.1371/journal.pbio.0020131 15045029 PMC374244

[B81] Kougnassoukou Tchara P. E. Filippakopoulos P. Lambert J. P. (2019). Emerging tools to investigate bromodomain functions. *Methods* 10.1016/j.ymeth.2019.11.003 [Epub ahead of print]. 31726225

[B82] Kuo M. H. Allis C. D. (1998). Roles of histone acetyltransferases and deacetylases in gene regulation. *Bioessays* 20 615–626. 10.1002/(sici)1521-1878(199808)20:8<615::aid-bies4>3.0.co;2-h 9780836

[B83] Lalonde M. E. Cheng X. Cote J. (2014). Histone target selection within chromatin: an exemplary case of teamwork. *Genes Dev.* 28 1029–1041. 10.1101/gad.236331.113 24831698 PMC4035532

[B84] Lee H. Z. Kwitkowski V. E. Del Valle P. L. Ricci M. S. Saber H. Habtemariam B. A. (2015). FDA approval: belinostat for the treatment of patients with relapsed or refractory peripheral T-cell lymphoma. *Clin. Cancer Res.* 21 2666–2670. 10.1158/1078-0432.ccr-14-3119 25802282

[B85] Lewis P. W. Beall E. L. Fleischer T. C. Georlette D. Link A. J. Botchan M. R. (2004). Identification of a *Drosophila* Myb-E2F2/RBF transcriptional repressor complex. *Genes Dev.* 18 2929–2940. 10.1101/gad.1255204 15545624 PMC534653

[B86] Li Y. Li H. Sui M. Li M. Wang J. Meng Y. (2019). Fungal acetylome comparative analysis identifies an essential role of acetylation in human fungal pathogen virulence. *Commun. Biol.* 2:154. 10.1038/s42003-019-0419-1 PMC6494858 31069264

[B87] Li Y. Wen H. Xi Y. Tanaka K. Wang H. Peng D. (2014). AF9 YEATS domain links histone acetylation to DOT1L-mediated H3K79 methylation. *Cell* 159 558–571. 10.1016/j.cell.2014.09.049 25417107 PMC4344132

[B88] Lin R. Leone J. W. Cook R. G. Allis C. D. (1989). Antibodies specific to acetylated histones document the existence of deposition- and transcription-related histone acetylation in *Tetrahymena*. *J. Cell Biol.* 108 1577–1588. 10.1083/jcb.108.5.1577 2654136 PMC2115542

[B89] Liu Y. Taverna S. D. Muratore T. L. Shabanowitz J. Hunt D. F. Allis C. D. (2007). RNAi-dependent H3K27 methylation is required for heterochromatin formation and DNA elimination in *Tetrahymena*. *Genes Dev.* 21 1530–1545. 10.1101/gad.1544207 17575054 PMC1891430

[B90] Luger K. Mader A. W. Richmond R. K. Sargent D. F. Richmond T. J. (1997). Crystal structure of the nucleosome core particle at 2.8 A resolution. *Nature* 389 251–260. 9305837 10.1038/38444

[B91] Martindale D. W. Allis C. D. Bruns P. J. (1982). Conjugation in *Tetrahymena thermophila*. A temporal analysis of cytological stages. *Exp. Cell Res.* 140 227–236. 10.1016/0014-4827(82)90172-0 7106201

[B92] Martindale D. W. Allis C. D. Bruns P. J. (1985). RNA and protein synthesis during meiotic prophase in *Tetrahymena thermophila*. *J. Protozool.* 32 644–649. 10.1111/j.1550-7408.1985.tb03094.x 2415700

[B93] Masumoto H. Hawke D. Kobayashi R. Verreault A. (2005). A role for cell-cycle-regulated histone H3 lysine 56 acetylation in the DNA damage response. *Nature* 436 294–298. 10.1038/nature03714 16015338

[B94] Miao J. Fan Q. Cui L. Li X. Wang H. Ning G. (2010). The MYST family histone acetyltransferase regulates gene expression and cell cycle in malaria parasite Plasmodium falciparum. *Mol. Microbiol.* 78 883–902. 10.1111/j.1365-2958.2010.07371.x 20807207 PMC2978264

[B95] Miao J. Lawrence M. Jeffers V. Zhao F. Parker D. Ge Y. (2013). Extensive lysine acetylation occurs in evolutionarily conserved metabolic pathways and parasite-specific functions during Plasmodium falciparum intraerythrocytic development. *Mol. Microbiol.* 89 660–675. 10.1111/mmi.12303 23796209 PMC3757501

[B96] Miao W. Xiong J. Bowen J. Wang W. Liu Y. Braguinets O. (2009). Microarray analyses of gene expression during the *Tetrahymena thermophila* life cycle. *PLoS One* 4:e4429. 10.1371/journal.pone.0004429 19204800 PMC2636879

[B97] Morrison A. J. Shen X. (2009). Chromatin remodelling beyond transcription: the INO80 and SWR1 complexes. *Nat. Rev. Mol. Cell Biol.* 10 373–384. 10.1038/nrm2693 19424290 PMC6103619

[B98] Nabeel-Shah S. Ashraf K. Saettone A. Garg J. Derynck J. Lambert J. P. (2020). Nucleus-specific linker histones Hho1 and Mlh1 form distinct protein interactions during growth, starvation and development in *Tetrahymena thermophila*. *Sci. Rep.* 10:168. 10.1038/s41598-019-56867-0 PMC6957481 31932604

[B99] Nagarajan P. Ge Z. Sirbu B. Doughty C. Agudelo Garcia P. A. Schlederer M. (2013). Histone acetyl transferase 1 is essential for mammalian development, genome stability, and the processing of newly synthesized histones H3 and H4. *PLoS Genet.* 9:e1003518. 10.1371/journal.pgen.1003518 23754951 PMC3675013

[B100] Nguyen H. H. T. Yeoh L. M. Chisholm S. A. Duffy M. F. (2020). Developments in drug design strategies for bromodomain protein inhibitors to target *Plasmodium falciparum* parasites. *Expert Opin. Drug Discov.* 15 415–425. 10.1080/17460441.2020.1704251 31870185

[B101] No Author (2018). Chasing histone biology from sea urchins to yeast. *Cell* 175 27–29. 10.1016/j.cell.2018.08.008 30217362

[B102] Ohba R. Steger D. J. Brownell J. E. Mizzen C. A. Cook R. G. Cote J. (1999). A novel H2A/H4 nucleosomal histone acetyltransferase in *Tetrahymena thermophila*. *Mol. Cell. Biol.* 19 2061–2068. 10.1128/mcb.19.3.2061 10022893 PMC83999

[B103] Orias E. Cervantes M. D. Hamilton E. P. (2011). *Tetrahymena thermophila*, a unicellular eukaryote with separate germline and somatic genomes. *Res. Microbiol.* 162 578–586. 10.1016/j.resmic.2011.05.001 21624459 PMC3132220

[B104] Orias E. Flacks M. (1975). Macronuclear genetics of *Tetrahymena*. I. Random distribution of macronuclear genecopies in pyriformis, T., syngen 1. *Genetics* 79 187–206. 805746 10.1093/genetics/79.2.187 PMC1213266

[B105] Papamichos-Chronakis M. Watanabe S. Rando O. J. Peterson C. L. (2011). Global regulation of H2A.Z localization by the INO80 chromatin-remodeling enzyme is essential for genome integrity. *Cell* 144 200–213. 10.1016/j.cell.2010.12.021 21241891 PMC3035940

[B106] Parker K. Maxson J. Mooney A. Wiley E. A. (2007). Class I histone deacetylase Thd1p promotes global chromatin condensation in *Tetrahymena thermophila*. *Eukaryot. Cell* 6 1913–1924. 10.1128/ec.00217-07 17715364 PMC2043386

[B107] Parthun M. R. (2007). Hat1: the emerging cellular roles of a type B histone acetyltransferase. *Oncogene* 26 5319–5328. 10.1038/sj.onc.1210602 17694075

[B108] Parthun M. R. (2013). Histone acetyltransferase 1: more than just an enzyme? *Biochim. Biophys. Acta* 1819 256–263. 10.1016/j.bbagrm.2011.07.006 24459728 PMC3206209

[B109] Parthun M. R. Widom J. Gottschling D. E. (1996). The major cytoplasmic histone acetyltransferase in yeast: links to chromatin replication and histone metabolism. *Cell* 87 85–94. 10.1016/s0092-8674(00)81325-2 8858151

[B110] Poli J. Gasser S. M. Papamichos-Chronakis M. (2017). The INO80 remodeller in transcription, replication and repair. *Philos. Trans. R. Soc. Lond. B Biol. Sci.* 372:20160290. 10.1098/rstb.2016.0290 28847827 PMC5577468

[B111] Recht J. Tsubota T. Tanny J. C. Diaz R. L. Berger J. M. Zhang X. (2006). Histone chaperone Asf1 is required for histone H3 lysine 56 acetylation, a modification associated with S phase in mitosis and meiosis. *Proc. Natl. Acad. Sci. U.S.A.* 103 6988–6993. 10.1073/pnas.0601676103 16627621 PMC1459006

[B112] Ren Q. Gorovsky M. A. (2001). Histone H2A.Z acetylation modulates an essential charge patch. *Mol. Cell.* 7 1329–1335. 10.1016/s1097-2765(01)00269-6 11430834

[B113] Richman R. Chicoine L. G. Collini M. P. Cook R. G. Allis C. D. (1988). Micronuclei and the cytoplasm of growing *Tetrahymena* contain a histone acetylase activity which is highly specific for free histone H4. *J. Cell Biol.* 106 1017–1026. 10.1083/jcb.106.4.1017 3360847 PMC2114999

[B114] Richters A. Koehler A. N. (2017). Epigenetic modulation using small molecules - targeting histone acetyltransferases in disease. *Curr. Med. Chem.* 24 4121–4150. 28240169 10.2174/0929867324666170223153115

[B115] Ruhl D. D. Jin J. Cai Y. Swanson S. Florens L. Washburn M. P. (2006). Purification of a human SRCAP complex that remodels chromatin by incorporating the histone variant H2A.Z into nucleosomes. *Biochemistry* 45 5671–5677. 10.1021/bi060043d 16634648

[B116] Sadasivam S. DeCaprio J. A. (2013). The DREAM complex: master coordinator of cell cycle-dependent gene expression. *Nat. Rev. Cancer* 13 585–595. 10.1038/nrc3556 23842645 PMC3986830

[B117] Saettone A. Garg J. Lambert J. P. Nabeel-Shah S. Ponce M. Burtch A. (2018). The bromodomain-containing protein Ibd1 links multiple chromatin-related protein complexes to highly expressed genes in *Tetrahymena thermophila*. *Epigenet. Chromatin* 11:10. 10.1186/s13072-018-0180-6 PMC5844071 29523178

[B118] Saettone A. Nabeel-Shah S. Garg J. Lambert J. P. Pearlman R. E. Fillingham J. (2019a). Functional proteomics of nuclear proteins in *Tetrahymena thermophila*: a review. *Genes* 10:333. 10.3390/genes10050333 31052454 PMC6562869

[B119] Saettone A. Ponce M. Nabeel-Shah S. Fillingham J. (2019b). RACS: rapid analysis of ChIP-Seq data for contig based genomes. *BMC Bioinformatics* 20:533. 10.1186/s12859-019-3100-2 31664892 PMC6819487

[B120] Saha A. Wittmeyer J. Cairns B. R. (2006). Chromatin remodelling: the industrial revolution of DNA around histones. *Nat. Rev. Mol. Cell Biol.* 7 437–447. 10.1038/nrm1945 16723979

[B121] Santos J. M. Josling G. Ross P. Joshi P. Orchard L. Campbell T. (2017). Red blood cell invasion by the malaria parasite is coordinated by the PfAP2-I transcription factor. *Cell Host Microbe* 21:e10. 10.1016/j.chom.2017.05.006 PMC5855115 28618269

[B122] Schulz D. Mugnier M. R. Paulsen E. M. Kim H. S. Chung C. W. Tough D. F. (2015). Bromodomain proteins contribute to maintenance of bloodstream form stage identity in the African Trypanosome. *PLoS Biol.* 13:e1002316. 10.1371/journal.pbio.1002316 26646171 PMC4672894

[B123] Shen X. Yu L. Weir J. W. Gorovsky M. A. (1995). Linker histones are not essential and affect chromatin condensation *in vivo*. *Cell* 82 47–56. 10.1016/0092-8674(95)90051-9 7606784

[B124] Shilatifard A. (2012). The COMPASS family of histone H3K4 methylases: mechanisms of regulation in development and disease pathogenesis. *Annu. Rev. Biochem.* 81 65–95. 10.1146/annurev-biochem-051710-134100 22663077 PMC4010150

[B125] Slade K. M. Freggiaro S. Cottrell K. A. Smith J. J. Wiley E. A. (2011). Sirtuin-mediated nuclear differentiation and programmed degradation in *Tetrahymena*. *BMC Cell Biol.* 12:40. 10.1186/1471-2121-12-40 21933443 PMC3191509

[B126] Smith J. J. Torigoe S. E. Maxson J. Fish L. C. Wiley E. A. (2008). A class II histone deacetylase acts on newly synthesized histones in *Tetrahymena*. *Eukaryot. Cell* 7 471–482. 10.1128/ec.00409-07 18178773 PMC2268513

[B127] Sobel R. E. Cook R. G. Perry C. A. Annunziato A. T. Allis C. D. (1995). Conservation of deposition-related acetylation sites in newly synthesized histones H3 and H4. *Proc. Natl. Acad. Sci. U.S.A.* 92 1237–1241. 10.1073/pnas.92.4.1237 7862667 PMC42674

[B128] Somech R. Izraeli S. Simson A. J. (2004). Histone deacetylase inhibitors–a new tool to treat cancer. *Cancer Treat. Rev.* 30 461–472. 10.1016/j.ctrv.2004.04.006 15245778

[B129] Stargell L. A. Bowen J. Dadd C. A. Dedon P. C. Davis M. Cook R. G. (1993). Temporal and spatial association of histone H2A variant hv1 with transcriptionally competent chromatin during nuclear development in *Tetrahymena thermophila*. *Genes Dev.* 7 2641–2651. 10.1101/gad.7.12b.2641 8276246

[B130] Stover N. A. Krieger C. J. Binkley G. Dong Q. Fisk D. G. Nash R. (2006). *Tetrahymena* genome database (TGD): a new genomic resource for *Tetrahymena thermophila* research. *Nucleic Acids Res.* 34 D500–D503. 16381920 10.1093/nar/gkj054 PMC1347417

[B131] Sui L. Zhang S. Huang R. Li Z. (2020). HDAC11 promotes meiotic apparatus assembly during mouse oocyte maturation via decreasing H4K16 and alpha-tubulin acetylation. *Cell Cycle* 19 354–362. 10.1080/15384101.2019.1711315 31910069 PMC7028157

[B132] Taverna S. D. Coyne R. S. Allis C. D. (2002). Methylation of histone h3 at lysine 9 targets programmed DNA elimination in *Tetrahymena*. *Cell* 110 701–711. 10.1016/s0092-8674(02)00941-8 12297044

[B133] Taverna S. D. Ueberheide B. M. Liu Y. Tackett A. J. Diaz R. L. Shabanowitz J. (2007). Long-distance combinatorial linkage between methylation and acetylation on histone H3 N termini. *Proc. Natl. Acad. Sci. U.S.A.* 104 2086–2091. 10.1073/pnas.0610993104 17284592 PMC1892956

[B134] Teo G. Liu G. Zhang J. Nesvizhskii A. I. Gingras A. C. Choi H. (2014). SAINTexpress: improvements and additional features in Significance Analysis of INTeractome software. *J. Proteomics* 100 37–43. 10.1016/j.jprot.2013.10.023 24513533 PMC4102138

[B135] Thatcher T. H. MacGaffey J. Bowen J. Horowitz S. Shapiro D. L. Gorovsky M. A. (1994). Independent evolutionary origin of histone H3.3-like variants of animals and *Tetrahymena*. *Nucleic Acids Res.* 22 180–186. 10.1093/nar/22.2.180 8121802 PMC307769

[B136] Tjeertes J. V. Miller K. M. Jackson S. P. (2009). Screen for DNA-damage-responsive histone modifications identifies H3K9Ac and H3K56Ac in human cells. *EMBO J.* 28 1878–1889. 10.1038/emboj.2009.119 19407812 PMC2684025

[B137] Travis G. H. Colavito-Shepanski M. Grunstein M. (1984). Extensive purification and characterization of chromatin-bound histone acetyltransferase from Saccharomyces cerevisiae. *J. Biol. Chem.* 259 14406–14412. 6389549

[B138] Vanagas L. Jeffers V. Bogado S. S. Dalmasso M. C. Sullivan W. J. Jr. Angel S. O. (2012). Toxoplasma histone acetylation remodelers as novel drug targets. *Expert Rev. Anti Infect. Ther.* 10 1189–1201. 10.1586/eri.12.100 23199404 PMC3581047

[B139] Vavra K. J. Allis C. D. Gorovsky M. A. (1982a). Regulation of histone acetylation in *Tetrahymena* macro- and micronuclei. *J. Biol. Chem.* 257 2591–2598. 7061439

[B140] Vavra K. J. Colavito-Shepanski M. Gorovsky M. A. (1982b). Histone acetylation and the deoxyribonuclease I sensitivity of the *Tetrahymena* ribosomal gene. *Biochemistry* 21 1772–1781. 10.1021/bi00537a012 6282318

[B141] Verreault A. Kaufman P. D. Kobayashi R. Stillman B. (1998). Nucleosomal DNA regulates the core-histone-binding subunit of the human Hat1 acetyltransferase. *Curr. Biol.* 8 96–108. 10.1016/s0960-9822(98)70040-5 9427644

[B142] Wai D. C. C. Szyszka T. N. Campbell A. E. Kwong C. Wilkinson-White L. E. Silva A. P. G. (2018). The BRD3 ET domain recognizes a short peptide motif through a mechanism that is conserved across chromatin remodelers and transcriptional regulators. *J. Biol. Chem.* 293 7160–7175. 10.1074/jbc.ra117.000678 29567837 PMC5949996

[B143] Wang J. Dixon S. E. Ting L. M. Liu T. K. Jeffers V. Croken M. M. (2014). Lysine acetyltransferase GCN5b interacts with AP2 factors and is required for *Toxoplasma gondii* proliferation. *PLoS Pathog.* 10:e1003830. 10.1371/journal.ppat.1003830 24391497 PMC3879359

[B144] Wang J. Gao S. Mostovoy Y. Kang Y. Zagoskin M. Sun Y. (2017). Comparative genome analysis of programmed DNA elimination in nematodes. *Genome Res.* 27 2001–2014. 10.1101/gr.225730.117 29118011 PMC5741062

[B145] Wang X. Wang S. Troisi E. C. Howard T. P. Haswell J. R. Wolf B. K. (2019). BRD9 defines a SWI/SNF sub-complex and constitutes a specific vulnerability in malignant rhabdoid tumors. *Nat. Commun.* 10:1881. 10.1038/s41467-019-09891-7 PMC6479050 31015438

[B146] Wei Y. Mizzen C. A. Cook R. G. Gorovsky M. A. Allis C. D. (1998). Phosphorylation of histone H3 at serine 10 is correlated with chromosome condensation during mitosis and meiosis in *Tetrahymena*. *Proc. Natl. Acad. Sci. U.S.A.* 95 7480–7484. 10.1073/pnas.95.13.7480 9636175 PMC22657

[B147] Wei Y. Yu L. Bowen J. Gorovsky M. A. Allis C. D. (1999). Phosphorylation of histone H3 is required for proper chromosome condensation and segregation. *Cell* 97 99–109. 10.1016/s0092-8674(00)80718-7 10199406

[B148] Wenkert D. Allis C. D. (1984). Timing of the appearance of macronuclear-specific histone variant hv1 and gene expression in developing new macronuclei of *Tetrahymena thermophila*. *J. Cell Biol.* 98 2107–2117. 10.1083/jcb.98.6.2107 6373790 PMC2113060

[B149] Wiley E. A. Myers T. Parker K. Braun T. Yao M. C. (2005). Class I histone deacetylase Thd1p affects nuclear integrity in *Tetrahymena thermophila*. *Eukaryot. Cell* 4 981–990. 10.1128/ec.4.5.981-990.2005 15879532 PMC1140101

[B150] Wiley E. A. Ohba R. Yao M. C. Allis C. D. (2000). Developmentally regulated rpd3p homolog specific to the transcriptionally active macronucleus of vegetative *Tetrahymena thermophila*. *Mol. Cell. Biol.* 20 8319–8328. 10.1128/mcb.20.22.8319-8328.2000 11046129 PMC102139

[B151] World Health Organization [WHO] (2010). *Working to Overcome the Global Impact of Neglected Tropical Diseases: First WHO Report on Neglected Tropical Diseases.* Geneva: World Health Organization.

[B152] Wurtele H. Tsao S. Lepine G. Mullick A. Tremblay J. Drogaris P. (2010). Modulation of histone H3 lysine 56 acetylation as an antifungal therapeutic strategy. *Nat. Med.* 16 774–780. 10.1038/nm.2175 20601951 PMC4108442

[B153] Xiong J. Yuan D. Fillingham J. S. Garg J. Lu X. Chang Y. (2011). Gene network landscape of the ciliate *Tetrahymena thermophila*. *PLoS One* 6:e20124. 10.1371/journal.pone.0020124 21637855 PMC3102692

[B154] Yan L. Wang L. Tian Y. Xia X. Chen Z. (2016). Structure and regulation of the chromatin remodeller ISWI. *Nature* 540 466–469. 10.1038/nature20590 27919072

[B155] Yang X. Wu X. Zhang J. Zhang X. Xu C. Liao S. (2017). Recognition of hyperacetylated N-terminus of H2AZ by TbBDF2 from *Trypanosoma brucei*. *Biochem. J.* 474 3817–3830. 10.1042/bcj20170619 29025975

[B156] Yao M. Chao J. Cheng C. (2015). “Programmed genome rearrangements in *Tetrahymena*,” in *Mobile DNA III* eds Craig N. Chandler M. Gellert M. Lambowitz A. Rice P. Sandmeyer S. (Washington, DC: ASM Press) 349–367. 10.1128/microbiolspec.MDNA3-0012-2014

[B157] Yu L. Gorovsky M. A. (1997). Constitutive expression, not a particular primary sequence, is the important feature of the H3 replacement variant hv2 in *Tetrahymena thermophila*. *Mol. Cell. Biol.* 17 6303–6310. 10.1128/mcb.17.11.6303 9343391 PMC232481

[B158] Zaware N. Zhou M. M. (2019). Bromodomain biology and drug discovery. *Nat. Struct. Mol. Biol.* 26 870–879. 10.1038/s41594-019-0309-8 31582847 PMC6984398

[B159] Zeng L. Zhang Q. Li S. Plotnikov A. N. Walsh M. J. Zhou M. M. (2010). Mechanism and regulation of acetylated histone binding by the tandem PHD finger of DPF3b. *Nature* 466 258–262. 10.1038/nature09139 20613843 PMC2901902

[B160] Zhang J. Yan G. Tian M. Ma Y. Xiong J. Miao W. (2018). A DP-like transcription factor protein interacts with E2fl1 to regulate meiosis in *Tetrahymena thermophila*. *Cell Cycle* 17 634–642. 10.1080/15384101.2018.1431595 29417875 PMC5969552

[B161] Zhao S. Xu W. Jiang W. Yu W. Lin Y. Zhang T. (2010). Regulation of cellular metabolism by protein lysine acetylation. *Science* 327 1000–1004. 20167786 10.1126/science.1179689 PMC3232675

